# Seedling survival simultaneously determined by conspecific, heterospecific, and phylogenetically related neighbors and habitat heterogeneity in a subtropical forest in Taiwan

**DOI:** 10.1002/ece3.8525

**Published:** 2022-01-12

**Authors:** Teng‐He Huang, Chun‐Lin Huang, Yi‐Ching Lin, I‐Fang Sun

**Affiliations:** ^1^ Department of Life Science Tunghai University Taichung Taiwan; ^2^ Department of Biology National Museum of Natural Science Taichung Taiwan; ^3^ Department of Natural Resources and Environmental Studies National Dong Hwa University Hualien Taiwan

**Keywords:** coexistence, Forest Dynamics Plot, generalized linear mixed models, negative density dependence, neighborhood effects, phylogenetics, seedling survival

## Abstract

Density dependence and habitat heterogeneity have been recognized as important driving mechanisms that shape the patterns of seedling survival and promote species coexistence in species‐rich forests. In this study, we evaluated the relative importance of density dependence by conspecific, heterospecific, and phylogenetically related neighbors and habitat heterogeneity on seedling survival in the Lienhuachih (LHC) Forest, a subtropical, evergreen forest in central Taiwan. Age‐specific effects of different variables were also studied. We monitored the fates of 1,642 newly recruited seedlings of woody plants within a 25‐ha Forest Dynamics Plot for 2 years. The effects of conspecific, heterospecific, and phylogenetically related neighbors and habitat heterogeneity on seedling survival were analyzed by generalized linear mixed models. Our results indicated that conspecific negative density dependence (CNDD) had a strong impact on seedling survival, and the effects of CNDD increased with seedling age. Heterospecific positive density dependence (HPDD) and phylogenetic positive density dependence (PPDD) had a significant influence on the survival of seedlings, and stronger HPDD and PPDD effects were detected for older seedlings. Furthermore, seedling survival differed among habitats significantly. Seedling survival was significantly higher in the plateau, high‐slope, and low‐slope habitats than in the valley. Overall, our results suggested that the effects of CNDD, HPDD, PPDD, and habitat heterogeneity influenced seedling survival simultaneously in the LHC subtropical forest, but their relative importance varied with seedling age. Such findings from our subtropical forest were slightly different from tropical forests, and these contrasting patterns may be attributed to differences in abiotic environments. These findings highlight the importance to incorporate phylogenetic relatedness, seedling age, and habitat heterogeneity when investigating the impacts of density dependence on seedling survival that may contribute to species coexistence in seedling communities.

## Graphical abstract

The relative importance of density dependence by conspecific, heterospecific, and phylogenetically related neighbors and habitat heterogeneity on seedling survival was evaluated in a subtropical forest. Our results revealed that the effects of CNDD, HPDD, PPDD, and habitat heterogeneity were prevalent in the LHC subtropical forest, and their relative importance varied among seedling ages. Our study highlights the importance to incorporate phylogenetic relatedness, seedling age, and habitat heterogeneity when investigating the impacts of density dependence on seedling survival that may contribute to species coexistence in seedling communities.

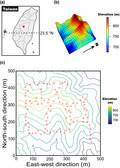

## 1 INTRODUCTION

Effects of negative density dependence (hereafter NDD) and habitat heterogeneity on seedling survival have been proposed over the past several decades as important driving mechanisms that explain species coexistence in species‐rich plant communities (Chesson, 2000; Keddy, 1992; Peters, 2003; Wright, 2002). Previous studies suggested that NDD and habitat heterogeneity may lead to spatial variation in seedling survival in various forests. Such variation may reduce likelihood of competitive exclusion and promote species coexistence (Bagchi et al., 2010; Chen et al., 2010; Comita et al., 2010; Harms et al., 2000; Johnson et al., 2012; Murphy et al., 2017). However, effects of density dependence and habitat heterogeneity have been studied separately in many previous studies (Du et al., 2017; Metz et al., 2010; Song et al., 2018), and their relative effects on seedling survival are seldom evaluated. Effects of density dependence and habitat heterogeneity may not be mutually exclusive (Lu et al., 2015; Pu et al., 2017). Their joint effects may differ from individual processes and should be studied simultaneously.

Conspecific negative density dependence (CNDD) is derived from host‐specific natural enemies, such as pathogens and herbivores (i.e., Janzen‐Connell effect (Janzen, 1970; Connell, 1971)) or intraspecific competition. CNDD might lead to a decrease in plant growth and an increase in mortality, hence lowers the strength of interspecific competition. As a result, competitive superior species may fail to establish at all suitable habitats, which frees space for other species. Such mechanisms reduce probability of competitive exclusion and facilitates species coexistence (Wright, 2002).

Although CNDD has been detected in many forest communities (Bagchi et al., 2010; Chen et al., 2010; Comita et al., 2010; Harms et al., 2000; Johnson et al., 2012; Murphy et al., 2017), effects of heterospecific neighbors on seedling survival are not clear from the literature. Both negative and positive effects have been demonstrated in previous studies (Comita & Hubbell, 2009; Johnson et al., 2014; Lu et al., 2015). The positive effect of heterospecific neighbors may result from the species herd protection hypothesis. This hypothesis posits that an increase in heterospecific crowding decreased encounter probabilities between focal individuals and their host‐specific enemies and, thus, survival of host plants increased (Peters, 2003; Wills & Green, 1995). Alternatively, a high density of heterospecific neighbors may have implied that the local habitat was suitable for seedling establishment and survival (Comita & Hubbell, 2009).

Categorizing neighbors into conspecific and heterospecific species, however, may be overly simplistic (Comita et al., 2014; Johnson et al., 2014; Piao et al., 2013). For example, intrinsic differences between heterospecific neighbors on a focal plant are ignored if we simply categorized different species of seedlings into one "heterospecific" group (Metz et al., 2010; Webb et al., 2006; Wu et al., 2016). Recent studies suggest that neighbors that were closely related phylogenetically had stronger negative impacts on the survival of focal plants than distant neighbors. Such effects are referred to as phylogenetic negative density dependence (PNDD) (Comita et al., 2018; Liu et al., 2012; Paine et al., 2011; Pu & Jin, 2018; Zhu et al., 2015). Meanwhile, phylogenetic positive density dependence on seedling survival (PPDD) was also observed in some studies (Cao et al., 2018; Lebrija‐Trejos et al., 2014; Wu et al., 2016; Zhu et al., 2015). More studies of phylogenetic density dependence are required to reconcile such inconsistent results.

Furthermore, seedling survival may vary with habitat heterogeneity. Seedling survival may be higher in favorable habitats than in unfavorable habitats. Spatial variation in seedling survival may serve as a filtering mechanism to regulate plant diversity (Bai et al., 2012; Lu et al., 2015; Metz, 2012; Pu et al., 2017). Some studies found that many species were aggregated and associated with certain habitats in tropical forests when forests were classified into different habitats based upon topographic factors (Comita et al., 2007; Metz, 2012). However, seedling aggregation in favorable habitats may enhance CNDD effects. To what extend do density dependence and habitat heterogeneity interact together to influence seedling survival is still unclear.

Effects of density dependence and habitat heterogeneity on seedling survival may not be constant during the life cycle of plants. Seedling age was an important factor that impacted dynamics of seedlings (Metz et al., 2010; Record et al., 2016; Zhu et al., 2015). For example, CNDD tended to be stronger on young seedlings during the early stages of plants (Comita et al., 2014; Zhu et al., 2015) because young seedlings lacked defense capacity and were more vulnerable to attacks by natural enemies (Wright, 2002). Alternatively, an increase in CNDD with seedling age may be caused by the pathogen accumulation over time or result from asymmetric intraspecific competition from conspecific trees (Benítez et al., 2013; Lin et al., 2012; Liu et al., 2012). Therefore, the increased effect of CNDD as seedlings age may promote diversity by enhancing the establishment of heterospecific species.

In this study, we used age‐specific seedling dynamics data from a subtropical evergreen forest in Taiwan to investigate the relative importance of CNDD, HPDD, PNDD, and habitat heterogeneity on seedling survival. We tracked the fates of multiple cohorts of newly recruited seedlings in relation to the density of their conspecific and heterospecific neighbors, and neighbor relatedness that was estimated by phylogenetic distances. In additional to biotic neighborhood, we also evaluated effects of habitat heterogeneity on seedling survival. Our specific questions were as follows: (1) What is the relative importance of density dependence by conspecific, heterospecific, and phylogenetically related neighbors and habitat heterogeneity on seedling survival? (2) Does the relative importance of conspecific, heterospecific, and phylogenetically related neighbors and habitat heterogeneity vary with seedling age?

## 2 METHODS

### 2.1 Study site

**FIGURE 1:**
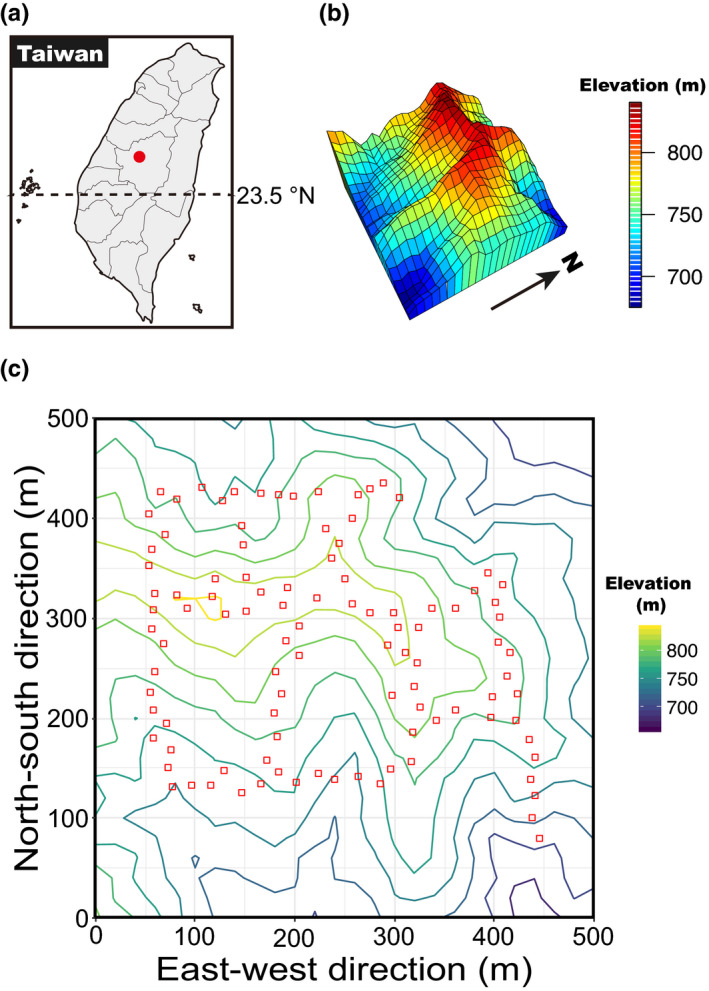
The location and map of the 25‐ha Lienhuachih Forest Dynamics Plot in central Taiwan. (a) The location of the Lienhuachih Forest Dynamics Plot is indicated with a red dot. (b) The 3D perspective map of the Lienhuachih Forest Dynamics Plot, and (c) the distribution of 102 seed trap stations (red hollow squares) within the 25‐ha Lienhuachih Forest Dynamics Plot

This study was conducted in the 25‐ha Forest Dynamics Plot in the Lienhuachih Experimental Forest (LHC; 23°54′49″N, 120°52′43″E), Nantou County, Taiwan (Figure 1a). The Lienhuachih Experimental Forest is managed by Taiwan Forestry Research Institute. According to meteorological records at the research station from 1997 to 2007, the climate at LHC is seasonal with a mean annual precipitation of 2,285 mm (Lu et al., 2008). Most precipitation occurred between May and August, and the dry season usually began in October and lasted through February of the next year. Monthly mean temperatures ranged from 14.8°C in January to 25.2°C in July, and mean annual temperature was 20.8°C.

The 25‐ha LHC Forest Dynamics Plot (500 m × 500 m) was established in 2008 following the survey protocol developed by the Forest Global Earth Observatory (ForestGeo) (Condit, 1998). The plot was very rugged with an elevational range from 667 m to 841 m asl. Slopes varied from 9° to 53° (Figure 1b). Every woody plant with diameter at breast height (DBH) ≥1 cm was tagged and mapped. Its species identity and DBH were also recorded. There were 153,484 individual trees that belonged to 46 families and 144 species recorded in the LHC plot. The vegetation type of the LHC plot was a subtropical, evergreen, broad‐leaved forest. The dominant species belonged to the families Fagaceae and Lauraceae (Chang et al., 2010).

### 2.2 Seedling census

One hundred and two census stations were established in August 2008 with an interval of 20 m along the trails and 4–10 m away from the trails (Figure 1c). Each station consisted of three 1 m^2^ seedling plots to monitor seedling dynamics, for a total of 306 seedling plots. In this study, we monitored the fate of each seedling from August 2008 to February 2011. Within each seedling plot, recruiting seedlings of woody plants <1 cm DBH were tagged, mapped, and identified to species. The survival status of all seedlings was checked, and new recruits were tagged every 6 months. We recorded the height of each seedling. Seedling height was measured from the ground to the base of the apical bud. In total, 3,997 seedlings that belonged to 71 species were identified. After excluding seedlings with unknown ages found during the first census in August 2008, we monitored the fate of 1,642 newly recruited seedlings.

### 2.3 Phylogenetic relatedness of seedlings

Phylogenetic relationships among 137 woody species were established in the LHC Forest Dynamics Plot by DNA barcoding. Three standard DNA barcoding loci (*matK*, *rbcL*, and *trnH*‐*psbA*) that were derived from the chloroplast genome were used. In this study, leaf tissues were collected from six individuals of each species and preserved through silica gel desiccation. DNA sequencing was conducted following Kress et al. (2009).

Community phylogeny that represented the 137 species of the LHC plot was constructed using three sequenced barcode loci (*matK*, *rbcL*, and *trnH*‐*psbA*) (Kress et al., 2009). The confidence of branches in a phylogenetic tree was estimated by maximum likelihood and rapid bootstrapping analysis and was computed using RAxML web servers (Stamatakis et al., 2008) in the CIPRES supercomputer cluster (www.phylo.org). Finally, the divergence time of an ultrametric tree was estimated using the r8s software package (Sanderson, 2003).

### 2.4 Data analysis

Generalized linear mixed‐effects models (GLMMs) were used to identify the factors that affected the survival of recruited seedlings. We developed eight candidate models to evaluate the relative importance of conspecific, heterospecific, and phylogenetically related neighbors and habitat heterogeneity on seedling survival. In the following analyses, we only included species that had seedlings found at ≥3 stations during the study period. We included the initial height (log transformed) of each seedling in the models because previous studies showed that seedling size exhibited significant effects on seedling survival (Lin et al., 2012; Wu et al., 2016). All seedlings, which included species with recruits that occurred at <3 census stations, in 1 m^2^ seedling plots were included in the analyses as seedling neighbors. In addition, we used all woody plants with DBH ≥1 cm that we tagged in 2008 as tree neighbors.

#### 2.4.1 Neighborhood variables

Eight density variables were calculated to estimate the neighborhood effects imposed by conspecific, heterospecific, or phylogenetically related species; four habitat types were used to estimate the effect of habitat quality on seedling survival (Table 1). We calculated the density of conspecific seedling neighbors (*S*_con_) and heterospecific seedling neighbors (*S*_het_) for the focal seedling within the 1 m^2^ seedling plots. The variables of conspecific tree neighbors (*T*_con_) and heterospecific tree neighbors (*T*_het_) were calculated within a 5 m radius of each focal individual. The influence of the tree neighbors was likely to be restricted within 5 m from the focal seedlings due to sharp topographic changes and relatively small tree crowns in LHC Forest Dynamics Plot. In addition, we ran the model for tree neighbors using three neighborhood radii: 5 m, 10 m, and 20 m away from focal seedlings. All neighborhood radii showed similar results qualitatively (Figure S1). Neighbor density indices of tree neighbors were estimated by the summed basal area (BA) of trees weighted by their distances between trees and the center of focal seedlings (Canham et al., 2004):

$$T_{\text{con}}\;\text{or}\;T_{\text{het}}=\sum_{i=1}^{N}\left(\frac{{\text{BA}}_{i}}{{\text{Distance}}_{i}}\right)$$

where N is the number of conspecific or heterospecific tree neighbors.

**TABLE 1:** The eight neighborhood variables and four habitat types examined in the study of seedling survival in the Lienhuachih Forest Dynamics Plot in Taiwan

Categories	Variables	Description
Conspecific neighbors	*S*_con_	Conspecific seedlings
	*T*_con_	Neighbor density index of conspecific trees
Heterogenetic neighbors	*S*_het_	Heterospecific seedlings
	*T*_het_	Neighbor density index of heterospecific trees
Phylogenetically related neighbors	*S*_APd′_	Seedling average phylodiversity
	*S*_NTPd′_	Seedling nearest‐taxon phylodiversity
	*T*_APd′_	Tree average phylodiversity
	*T*_NTPd′_	Tree nearest‐taxon phylodiversity
Habitat types	*H*_plateau_	Plateau habitats
	*H*_high‐slope_	High‐slope habitats
	*H*_low‐slope_	Low‐slope habitats
	*H*_valley_	Valley habitats

Note

Conspecific neighbors included the density of conspecific seedling neighbors (*S*_con_) and neighbor density index of heterospecific trees (*T*_con_). Heterospecific neighbors included the density of heterospecific seedling neighbors (*S*_het_) and neighbor density index of heterospecific trees (*T*_het_). Phylogenetically related neighbors included four phylogenetic diversity indices: relative average phylogenetic diversity between heterospecific seedling neighbors and focal seedlings (*S*_APd′_), relative nearest‐taxon phylodiversity between heterospecific seedling neighbors and focal seedlings (*S*_NTPd′_), relative average phylogenetic diversity between heterospecific tree neighbors and focal seedlings (*T*_APd′_), and relative nearest‐taxon phylodiversity between heterospecific tree neighbors and focal seedlings (*T*_NTPd′_). Habitat types included plateau (*H*_plateau_), high‐slope (*H*_high‐slope_), low‐slope (*H*_low‐slope_), and valley habitats (*H*_valley_).

Phylogenetic relatedness between every seedling and its neighbors was estimated by two variables, the average phylodiversity (APd′) and nearest‐taxon phylodiversity (NTPd′). These two variables were calculated as follows:

$${\text{Seedling APd}}^{\prime }{\;\text{or Tree APd}}^{\prime }=\frac{‐1({\text{MPD}}_{\text{obs}}‐\text{mean}\left({\text{MPD}}_{\text{null}}\right))}{\text{SD}({\text{MPD}}_{\text{null}})}$$

$${\text{Seedling NTPd}}^{\prime }{\;\text{or Tree NTPd}}^{\prime }=\frac{‐1({\text{NTD}}_{\text{obs}}‐\text{mean}\left({\text{NTD}}_{\text{null}}\right))}{\text{SD}({\text{NTD}}_{\text{null}})}$$

where the phylogenetic relatedness of MPD_obs_ and NTD_obs_ was calculated by the mean phylogenetic distance (MPD) and the nearest‐taxon distance (NTD) of all seedlings or adult neighbors to the focal seedling using branch lengths, respectively (Webb et al., 2006). Phylogenetic relatedness for seedling and tree neighbors was calculated within the 1 m^2^ seedling plots and 5 m radius, respectively. We obtained the expected phylogenetic distances using a null model to generate 999 random neighborhoods at a given species richness (MPD_null_ and NTD_null_) because phylogenetic distance varied with the species richness of the sample. The mean and standard deviation of the distribution of null model distances were combined with our observed phylogenetic distance to obtain a standard effect size, which indicated whether the focal seedling was more or less related to its neighbors than expected by the null model (Kraft et al., 2007; Webb, 2000; Webb et al., 2006). The average phylodiversity (APd′) was calculated based on the standard effect size of MPD. Nearest‐taxon phylodiversity (NTPd′) was calculated based on the standard effect size of NTD. If APd′ or NTPd′ was positive, it indicated that the neighbors were related more closely to the focal seedling than expected from the null model. In contrast, negative values of APd′ or NTPd′ suggested that the neighbors had a more distant phylogenetic relatedness to the focal seedling.

To incorporate temporal variation in seedling neighbors during the persistence period of each focal seedling, we calculated the effects of APd′ or NTPd′ (Table 1; *S*_APd′_ and *S*_NTPd′_) for each seedling at each census. Meanwhile, the seedling–adult phylogenetic relatedness of APd′ or NTPd′ for trees (*T*_APd′_ and *T*_NTPd′_) was only estimated once based on the 2008 census.

#### 2.4.2 Habitat classification

The habitat was classified into different types based upon three topographic variables and tree abundance. The three topographic variables of mean elevation, slope, and convexity were calculated from the elevation measurements taken from each intersection of 20 m × 20 m quadrats in the 25‐ha plot. Mean elevation was the average of elevations from four corners of the 20 m × 20 m quadrat. Slope was calculated by the mean angle from the horizontal of the entire quadrat (Harms et al., 2001). Convexity was the mean elevation of the focal quadrat minus the mean elevation of its eight surrounding quadrats. A multivariate regression tree analysis (MRT) was applied to classify habitats (Larsen & Speckman, 2004). Our MRT model selected convexity and slope to define four types of habitats (Figures S2 and S3). The four habitat types were plateau, high slope, low slope, and valley (Figures S2 and S3).

### 2.5 Statistical analysis

We used GLMMs to identify the factors that affected the survival of recruited seedlings. The model was used to quantify the overall fixed effects when spatial autocorrelation was nested within seedling plots (Dormann et al., 2007). The three 1 m^2^ seedling plots were regarded as three replicates at each census station. Seedling identity was included in the overall models as a random factor to accommodate repeated measurements of the same seedlings. Seedling response over the period of interest was regarded as binomial data (alive (1) or dead (0)) for the dependent variable. Seedling plots nested within each census station were incorporated in the models as random factors. In the analyses, density of seedling neighbors, neighbor density indices of adult trees, phylogenetic relatedness of seedling and tree neighbors, and habitat types were used for predictor variables in the models. We standardized continuous explanatory variables using a Z transformation (i.e., subtracting the mean values and dividing by the SD).

We constructed eight candidate models for seedling survival (Table 2). In the null model, seedling survival only correlated with the initial height of the focal seedling. To estimate the effects of biotic neighbors on seedling survival, we created conspecific and heterospecific models (Models 2 and 3, Table 2). To access the importance of phylogenetic relationships between the focal seedling and heterospecific neighborhood, we then constructed phylogenetic models that included APd′ and NTPd′ as variables (model 4, Table 2). We also considered the relationship between conspecific and heterospecific neighbors (model 5, Table 2), or we replaced the heterospecific neighbors into phylogenetically related neighbors (model 6, Table 2). Phylogenetically related neighbors may have had different effects compared with heterospecific neighbors, so we included all variables in the model to understand the relative importance of all variables (model 7, Table 2). Finally, we constructed a model with biotic neighbors and habitat types. The habitat was a categorical variable. In our analysis, the valley habitat was used as a baseline habitat for comparison (model 8, Table 2). In addition to the overall model, we constructed age‐specific models to evaluate the effect of seedling age. Eight candidate models with the same variables as the overall model were established for each age (age of 6 months, 12 months, 18 months, and 24 months). We used variance inflation factor (VIF) to check the multicollinearity between independent variables of our models. The results indicated that multicollinearity was not concerned in our models since the values of VIF ranged from 1.07 to 1.60. Furthermore, we also check residuals of GLMMs, there is no spatial autocorrelation for the full models.

**TABLE 2:** Description of the eight candidate models that explored the relationship among seedling survival, neighborhood effects, and habitat heterogeneity in the Lienhuachih Forest Dynamics Plot in Taiwan

Models		AIC	$R_{\text{mar}}^{2}$ (%)	$R_{\text{con}}^{2}$ (%)
1	Null model	4055.9	2.4	85.7
2	Conspecific	4034.7	7.5	86.2
3	Heterospecific	4025.3	9.4	86.4
4	Phylogenetic	4047.1	4.3	86.4
5	Conspecific + Heterospecific	4021.0	8.6	86.0
6	Conspecific + Phylogenetic	4015.7	9.0	86.6
7	Conspecific + Heterospecific + Phylogenetic	3999.3	10.9	86.6
8	Conspecific + Heterospecific + Phylogenetic + Habitat	3969.6*	16.1	87.3

Note

The most parsimonious model was chosen based on the smallest values of Akaike information criterion (AIC) (Labelled by *).

We used the Akaike information criterion (AIC) to select the most parsimonious models. The model with the smallest AIC value was selected. To compare the predictive capacity of GLMM models, marginal *R* ^2^ ($R_{\text{mar}}^{2}$) and conditional R^2^ ($R_{\text{con}}^{2}$) were developed by Nakagawa and Schielzeth (2013). $R_{\text{mar}}^{2}$ provided the proportion of variance explained by fixed effects, and $R_{\text{con}}^{2}$ accounted for total variance, which included both fixed and random effects. All analyses were performed using the statistical programming language R, Version 4.1.1 (R Core Team, 2021), and the packages ape, lme4, mvpart, picante, and vegan Bates et al., 2015; Kembel et al., 2010; Oksanen et al., 2020; Paradis et al., 2004).

## 3 RESULTS

### 3.1 The relative importance of conspecific, heterospecific, phylogenetically related neighbors, and habitat heterogeneity

**FIGURE 2:**
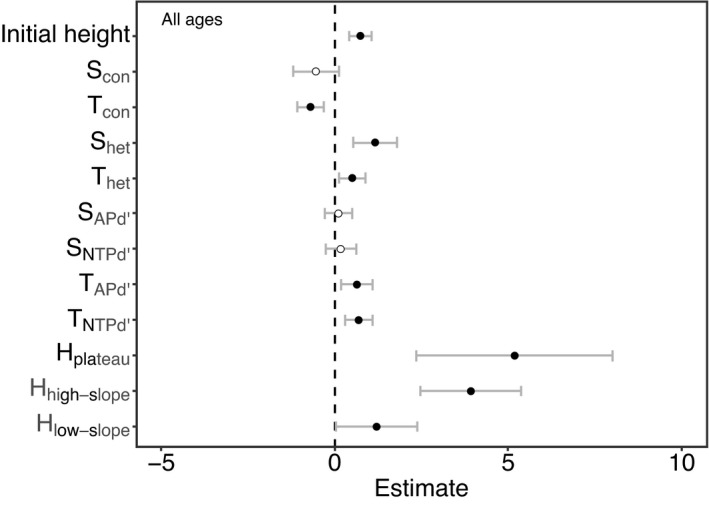
The coefficient estimates of the model and 95% confidence intervals of the variables between focal seedlings at different neighborhood scales and habitat types on the Lienhuachih Forest Dynamic Plot. Coefficient estimates above and below zero indicate positive and negative effects on seedling survival, respectively. The black circles indicate significant effects (*p* < .05), and white circles mean no significance

**FIGURE 3:**
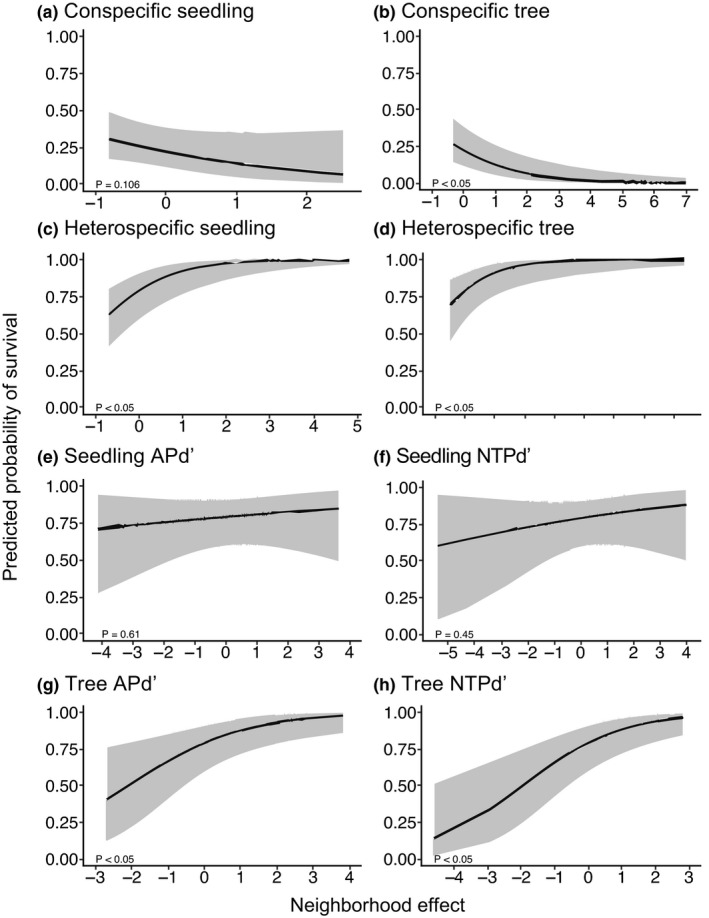
Predicted effects of conspecific, heterospecific, phylogenetically related neighbors, and habitat heterogeneity on the probability of survival for individuals of all ages in the Lienhuachih Forest, Taiwan. Lines show predictions based on model results with all independent variables assigned to their mean values

The full model that contained all the variables was selected as the most parsimonious model for seedlings of all ages combined (AIC = 3969.6; Table 2). In the model, 87.3% of the total variance was explained by the independent variables (Table 2). Fixed effects explained 16.1% of the variance (Table 2). This indicated that conspecific, heterospecific, and phylogenetically related neighbors and habitat heterogeneity all contributed to seedling survival. Survival of newly recruited seedlings was impacted significantly by conspecific tree neighbors (Table 3, Figure 2). The probability of seedling survival decreased when the conspecific neighbor density index increased (Figure 3b). In contrast, seedling survival was correlated positively with density of heterospecific seedlings and heterospecific neighbor density index (Figures 2, 3c,d). The variables of Tree APd′ and Tree NTPd′ related to phylogenetic relatedness of heterospecific tree neighbors had significantly positive impacts on seedling survival (Figure 2). The survival curves of Tree APd′ and Tree NTPd′ showed a similar pattern where probability of seedling survival increased with more closely related tree neighbors (Figure 3g,h). Moreover, seedling survival varied significantly among habitat types (Table 3, Figure 2). Positive parameter estimates indicated that seedlings survived significantly better in the plateau, high‐slope, and low‐slope habitats than in the valley habitat, which was treated as a baseline for comparison in this analysis (Figure 2).

**TABLE 3:** The results of GLMMs for seedling survival in the Lienhuachih Forest Dynamics Plot in Taiwan

Variables	Model 8		
	df	χ ^2^	*p*
Initial height	1	21.052	**<.05**
*S*_con_	1	2.618	.106
*T*_con_	1	13.102	**<.05**
*S*_het_	1	13.406	**<.05**
*T*_het_	1	7.043	**<.05**
*S*_APd′_	1	0.267	.606
*S*_NTPd′_	1	0.577	.448
*T*_APd′_	1	7.658	**<.05**
*T*_NTPd′_	1	11.602	**<.05**
Habitat	3	34.661	**<.05**

Note

Probability values less than 0.05 are shown in bold.

### 3.2 Relative importance of neighborhood variables and habitat heterogeneity for seedlings of different ages

**FIGURE 4:**
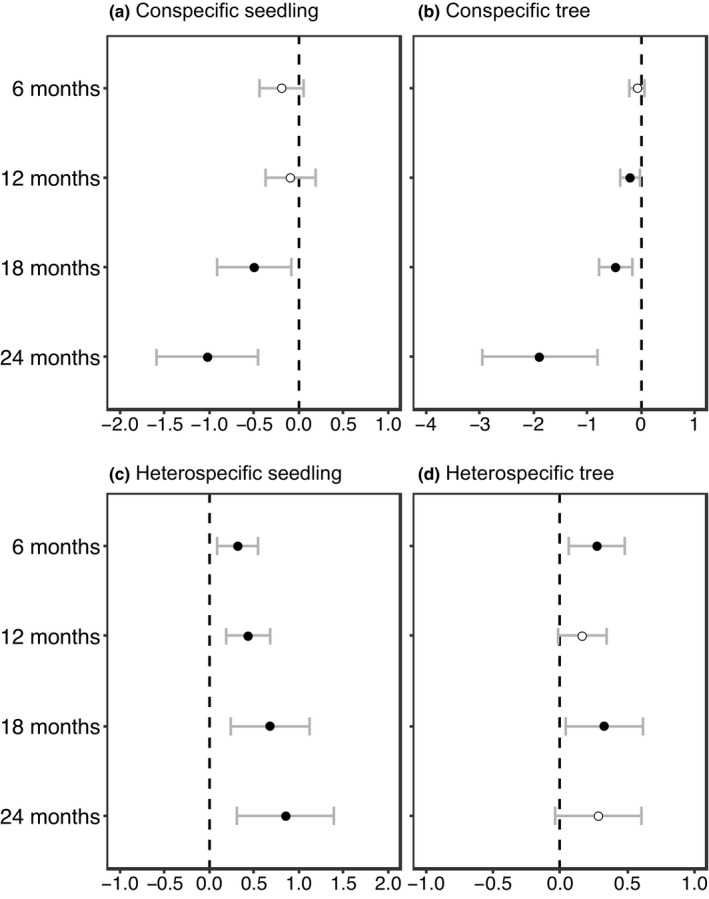
The coefficient estimates and 95% confidence intervals of the variables of conspecific and heterospecific neighbors on the Lienhuachih Forest Dynamic Plot for different seedling ages. Coefficient estimates above and below zero indicate positive and negative effects on seedling survival, respectively. The black circles indicate significant effects (*p* < .05) and white circles mean no significance

For seedlings of four different ages (6 months, 12 months, 18 months, and 24 months), the full model, which contained conspecific, heterospecific, and phylogenetically related neighbors and habitat heterogeneity, was identified as the most parsimonious model (Table 4). Significantly negative effects of conspecific seedling neighbors were detected for older seedlings (18 months and 24 months) (Table 5). Interestingly, effects of negative density dependence increased with seedling age (Table 5, Figure 4a). Conspecific tree neighbors also exhibited negative effects on focal seedlings (except for 6 months) (Table 5, Figure 4b). Contrary to conspecific neighborhood effects, heterospecific seedling neighbors had a positive impact on focal seedlings of four different ages, and the strength of the effect increased as seedlings aged (Table 5, Figure 4c). Heterospecific tree neighbors also showed positive density effects on focal seedlings of all ages (Table 5, Figure 4d).

**TABLE 4:** Values of Akaike information criterion (AIC) for the eight model classes for seedlings of four age classes (6 months, 12 months, 18 months, and 24 months) in the Lienhuachih Forest Dynamics Plot in Taiwan

Models	AIC			
	6 months (*N* = 1,556)	12 months (*N* = 1,298)	18 months (*N* = 733)	24 months (*N* = 630)
Null model: without neighborhood variables	2,038.0	1,673.0	924.9	718.9
Conspecific	2,035.4	1,667.2	910.6	671.3
Heterospecific	2,022.6	1,655.4	902.2	685.3
Phylogenetic	2,043.8	1,679.8	926.2	718.5
Conspecific + Heterospecific	2,024.2	1,656.7	900.6	663.3
Conspecific + Phylogenetic	2,028.2	1,661.3	889.0	646.2
Conspecific + Heterospecific + Phylogenetic	2,017.2	1,650.2	875.1	636.7
Conspecific + Heterospecific + Phylogenetic + Habitat	2,006.9*	1,633.6*	865.9*	627.8*

Note

The most parsimonious models were chosen based on the smallest AIC values (Labelled by *).

**TABLE 5:** Neighborhood effects on survival of seedlings of different ages in the Lienhuachih Forest Dynamics Plot in Taiwan

Variables^a^	6 months	12 months	18 months	24 months
Initial height	0.220*	0.264*	0.390*	0.225
*S*_con_	−0.191	−0.094	−0.498*	−1.019*
*T*_con_	−0.059	−0.198*	−0.468*	−1.889*
*S*_het_	0.319*	0.433*	0.679*	0.856*
*T*_het_	0.275*	0.164	0.327*	0.285
*S*_APd′_	−0.004	0.015	0.392*	0.458*
*S*_NTPd′_	0.060	0.078	0.029	−0.243
*T*_APd′_	0.221*	0.256*	0.267	0.465*
*T*_NTPd′_	0.194*	0.152	0.358*	0.489*
*H*_plateau_ ^b^	1.568*	1.556*	1.911*	1.798
*H*_high‐slope_ ^b^	1.005*	1.389*	1.459*	1.696*
*H*_low‐slope_ ^b^	0.311	0.452	0.482	0.935*

^a^ Values are coefficient estimates of fixed‐factor variables in a generalized linear mixed‐effects model. Variables from seedling neighbors were found within the same 1 m^2^ seedling plot as each focal seedling, and variables from tree neighbors were calculated within 5 m radius by each focal seedling. See Methods for a description of the generalized linear mixed‐effects model. Significant coefficients are denoted by the symbol * (*p* < .05). Coefficient estimates >0 have a positive relationship with increased survival, and coefficient estimates <0 have a negative relationship with survival. Positive effects (Coefficient estimates >0) indicate better survival with increased phylogenetic clustering; and negative effects (Coefficient estimates <0) indicate survival decreased when neighbors were more closely related.

^b^ Habitat types are categories data of the generalized linear mixed‐effects model. All estimate values for plateau, high‐slope, and low‐slope habitats were compared over the baseline of valley habitats.

**FIGURE 5:**
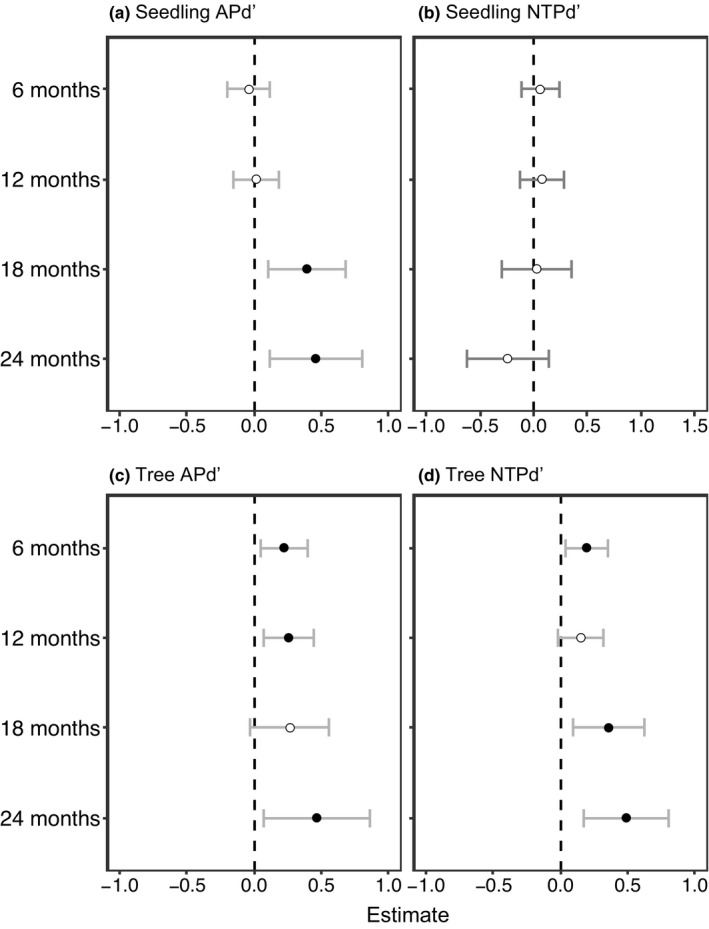
The coefficient estimates and 95% confidence intervals of the variables of phylogenetic neighbors on the Lienhuachih Forest Dynamic Plot for different seedling ages. Coefficient estimates above and below zero indicate positive and negative effects on seedling survival, respectively. The black circles indicate significant effects (*p* < .05), and white circles mean no significance

Phylogenetic relatedness of heterospecific neighbors exhibited a significant effect on seedling survival, but the strength of APd′ and NTPd′ effects varied with seedling age (Table 5, Figure 5). Phylogenetic relatedness among seedling neighbors only demonstrated significant effects for older seedlings. Seedling APd′ impacted seedling survival positively when focal seedlings were 18 months and 24 months old (Figure 5a). In addition, both APd′ and NTPd′ of tree neighbors affected seedling survival positively. These positive impacts indicated that focal seedlings surrounded by closely related tree neighbors were more likely to survive (Figure 5c,d).

**FIGURE 6:**
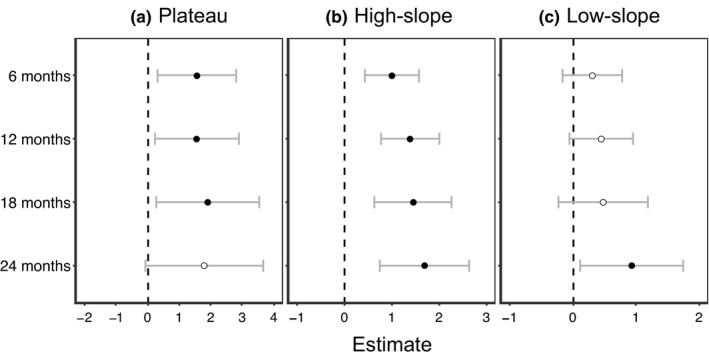
The coefficient estimates and 95% confidence intervals of the variables of different habitat types (baseline of valley habitat) on the Lienhuachih Forest Dynamic Plot for different seedling ages. Coefficient estimates above and below zero indicate positive and negative effects on seedling survival, respectively. The black circles indicate significant effects (*p* < .05), and white circles mean no significance

Seedling survival of four different ages varied significantly among habitats (Table 5). Compared with the baseline habitat of valley, the plateau and high‐slope habitats exhibited positive effects on seedling survival of all ages (Table 5, Figure 6). Meanwhile, significantly positive effects of plateau and high‐slope habitats were detected on seedlings of all ages except for 24 months in the plateau habitat (Table 5, Figure 6).

## 4 DISCUSSION

### 4.1 What is the relative importance of density dependence by conspecific, heterospecific, phylogenetically related neighbors and habitat heterogeneity on seedling survival?

Our results revealed that all of the factors examined in this study, which included CNDD, HPDD, PPDD, and habitat heterogeneity, contributed significantly to seedling survival. Among the four factors, conspecific tree neighbors had a strong and significant negative effect on seedling survival. In contrast, seedling survival was enhanced by density of heterospecific neighbors and phylogenetically closely neighbors. Finally, seedling survival differed significantly among habitats.

Many studies found seedling survival was influenced by CNDD in both tropical and temperate forests (Chen et al., 2018; Comita et al., 2014; Piao et al., 2013; Wu et al., 2016). Our results were consistent with those studies where significant CNDD effects were detected. CNDD effects may arise from the Janzen–Connell effects or intraspecific competition. It is widely accepted that seedlings may suffer from density‐dependent mortality caused by species‐specific natural enemies, such as pathogens and herbivores (Connell, 1971; Janzen, 1970). The phenomenon of “damping‐off” mortality of a large portion of seedlings was considered as evidence that supports the Janzen–Connell effects (Augspurger, 1984; Bayandala et al., 2017; Hood et al., 2004). In our study, none of the seedlings exhibited symptoms of damping‐off diseases or pathogen infection. Therefore, CNDD may be more likely to arise from intraspecific competition instead of the Janzen–Connell effects in the LHC forest.

Phylogenetic density dependence can be regarded as an extension of heterospecific density dependence (Liu et al., 2012; Metz et al., 2010; Webb et al., 2006). Our study indicated that the most parsimonious model included effects of HPDD from seedling and tree neighbors and PPDD from tree neighbors. The significant HPDD effects found in our study were similar to several studies conducted in subtropical and temperate forests (Chen et al., 2010; Du et al., 2017; Lu et al., 2015).

In addition, our study also detected strong PPDD effects on seedling survival from tree neighbors. Positive APd′ or NTPd′ relationships between phylogenetic similarity of neighbors and focal seedling survival may have occurred due to plant–microorganism relationships. Plant species in the same genus or family tend to share symbiotic partners in the soil (Aryal et al., 2021; Losos, 2008; Wu et al., 2018). Arbuscular mycorrhizas (AM) and ectomycorrhizal (ECM) colonize in the root tissues and facilitate the transport of nutrients and water. Liang et al. (2020) demonstrated that local dominance of trees was maintained by ECM fungal networks. Young seedlings benefited from common mycorrhizal networks. Especially, different species of seedlings may be connected to one another by a common mycelium of the same species of ECM fungi (Simard et al., 1997). The extensive underground mycorrhizal networks created by ECM fungi may have enhanced seedling survival of tree species from a small number of families that were associated with ECM fungi. Therefore, the strong PPDD effects may have reflected the phylogenetic connections with the ECM fungi.

In our study, seedling survival differed significantly among habitats, in which seedling survival was lowest in the valley habitat. A local soil survey indicated that soil moisture was significantly higher in the valley habitat than the other three habitats (Figure S4). Such patterns contradicted to previous studies where seedling survival was higher in moist habitats (Brenes‐Arguedas et al., 2009; Engelbrecht & Kursar, 2003). In LHC forest, soil moisture may not be a critical limiting factor since the annual precipitation is over 2,229 mm. Instead, light may play a more important role than soil moisture, especially for seedlings occurred in the valley habitat where trees are much taller than those in the plateau and slopes. Tall trees cast deep shade and reduced light availability in the understory. This low light environment in the valley habitat may lead to a decrease in seedling survival. In contrast to the valley habitat, plateaus, high slopes, and low slopes may have intermediate levels of soil moisture and light conditions that may optimize seedling survival in these three habitats. In addition, seedling survival in the plateaus slopes may be promoted further by PPDD as a result of plant–microorganism associations.

### 4.2 Does the relative importance of neighborhood effects and habitat heterogeneity vary with seedling age?

Overall, our results showed that the relative importance of each set of variables changed as seedlings aged, which likely reflected a shift in the relative importance of different biotic and abiotic interactions over the lifetime of plants. Our results revealed that negative effects of conspecific seedlings and tree neighbors increased as focal seedlings aged. In contrast, increased positive effects with increasing seedling age was only observed in heterospecific seedling neighbors. The positive effects of phylogenetic relatedness increased with increasing seedling age, which indicated that seedlings survived better when they were near closely related neighbors. Furthermore, seedlings in the plateaus, high‐slope, and low‐slope habitats continued to survive better than in the valley habitats throughout 6–24 months. This suggested that HPDD, PPDD, and habitat heterogeneity persisted as seedlings aged.

It has long been recognized that the effect of CNDD was strongest during early life stages (Augspurger, 1983; Bell et al., 2006; Liu et al., 2012; Mangan et al., 2010). The prevailing interpretation has been those pathogens, which caused damping‐off disease in young seedlings, were agents of density‐dependent mortality in tropical forests (Augspurger, 1984; Bayandala et al., 2017; Hood et al., 2004). One may expect CNDD to decrease as seedlings aged because if strong CNDD reduced the density of conspecific neighbors in early life stages, CNDD will be reduced at later life stages (Comita et al., 2007; Newbery & Stoll, 2013; Piao et al., 2013; Zhu et al., 2015). However, in this study, we found the opposite in that the effects of CNDD on seedlings and tree neighbors increased when focal seedlings aged. Our contrary observations may have resulted from the different causes of CNDD. As stated earlier, intraspecific competition was suggested to be the main cause of CNDD in this forest. As seedlings aged and became bigger, the demand for limited resources increased, thus, the strength of intraspecific competition increased. This may have resulted in higher CNDD as seedlings aged.

## 5 CONCLUSIONS

In summary, this study highlights that integrated phylogenetic relatedness, seedling age, and habitat heterogeneity were important when investigating the elements that contribute to species coexistence in tree communities. Our results revealed that the effects of CNDD, HPDD, PPDD, and habitat heterogeneity were prevalent in the LHC subtropical forest, and their relative importance varied among seedling ages. Our CNDD results suggested that negative conspecific effects were driven mainly by intraspecific competition rather than the Janzen–Connell effects in the seedling stage. Furthermore, the results implied that the underlying mechanisms of HPDD and PPDD effects may have resulted from habitat filtering and the networks of ectomycorrhizal fungal communities in this subtropical forest.

Furthermore, the strength of neighborhood effects in this subtropical forest was slightly different from tropical forests, and these contrasting patterns may be attributed to topographic heterogeneity. Our results suggested that studies in subtropical forests have failed to take habitat heterogeneity into account, and they may also have mischaracterized the role of biotic neighbors in fluctuating plant communities. Therefore, we suggested that future studies should take neighborhood effects and habitat heterogeneity into account simultaneously and also test for the effects in different seedling ages.

## CONFLICT OF INTEREST

None declared.

## AUTHOR CONTRIBUTIONS

**Teng‐He Huang:** Conceptualization (equal); data curation (equal); formal analysis (lead); investigation (lead); methodology (lead); writing – original draft (lead). **Chun‐Lin Huang:** Data curation (equal); formal analysis (supporting); methodology (supporting); supervision (equal). **Yiching Lin:** Conceptualization (supporting); formal analysis (supporting); methodology (supporting); project administration (supporting); resources (supporting); supervision (equal); validation (equal); writing – original draft (supporting); writing – review and editing (equal). **Ifang Sun:** Conceptualization (supporting); data curation (equal); funding acquisition (lead); methodology (supporting); project administration (lead); resources (lead); supervision (equal); validation (equal); writing – original draft (supporting); writing – review and editing (equal).

## Supporting information

Supplementary Material

## Data Availability

Data from this manuscript were archived in the Dryad Data Repository at https://doi.org/10.5061/dryad.4mw6m909m.

## References

[ece38525-bib-0001] Aryal, P. , Meiners, S. J. , & Carlsward, B. S. (2021). Ectomycorrhizae determine chestnut seedling growth and drought response. Agroforestry Systems, 95, 1251–1260. 10.1007/s10457-020-00488-4

[ece38525-bib-0002] Augspurger, C. K. (1983). Seed dispersal of the tropical tree, *Platypodium elegans*, and the escape of its seedlings from fungal pathogens. Journal of Ecology, 71, 759–771. 10.2307/2259591

[ece38525-bib-0003] Augspurger, C. K. (1984). Seedling survival of tropical tree species: Interactions of dispersal distance, light‐gaps, and pathogens. Ecology, 65, 1705–1712. 10.2307/1937766

[ece38525-bib-0004] Bagchi, R. , Swinfield, T. , Gallery, R. E. , Lewis, O. T. , Gripenberg, S. , Narayan, L. , & Freckleton, R. P. (2010). Testing the Janzen‐Connell mechanism: Pathogens cause overcompensating density dependence in a tropical tree. Ecology Letters, 13, 1262–1269. 10.1111/j.1461-0248.2010.01520.x 20718845

[ece38525-bib-0005] Bai, X. , Queenborough, S. , Wang, X. , Zhang, J. , Li, B. , Yuan, Z. , Xing, D. , Lin, F. , Ye, J. , & Hao, Z. (2012). Effects of local biotic neighbors and habitat heterogeneity on tree and shrub seedling survival in an old‐growth temperate forest. Oecologia, 170, 755–765. 10.1007/s00442-012-2348-2 22644047

[ece38525-bib-0006] Bates, D. , Mächler, M. , Bolker, B. , & Walker, S. (2015). Fitting linear mixed‐effects models using lme4. Journal of Statistical Software, 67, 1–48. 10.18637/jss.v067.i01

[ece38525-bib-0007] Bayandala , Masaka, K. , & Seiwa, K. (2017). Leaf diseases drive the Janzen‐Connell mechanism regardless of light conditions: A 3‐year field study. Oecologia, 183, 191–199. 10.1007/s00442-016-3757-4 27785649

[ece38525-bib-0008] Bell, T. , Freckleton, R. P. , & Lewis, O. T. (2006). Plant pathogens drive density‐dependent seedling mortality in a tropical tree. Ecology Letters, 9, 569–574. 10.1111/j.1461-0248.2006.00905.x 16643302

[ece38525-bib-0009] Benítez, M.‐S. , Hersh, M. H. , Vilgalys, R. , & Clark, J. S. (2013). Pathogen regulation of plant diversity via effective specialization. Trends in Ecology & Evolution, 28, 705–711. 10.1016/j.tree.2013.09.005 24091206

[ece38525-bib-0010] Brenes‐Arguedas, T. , Coley, P. D. , & Kursar, T. A. (2009). Pests vs. drought as determinants of plant distribution along a tropical rainfall gradient. Ecology, 90, 1751–1761. 10.1890/08-1271.1 19694125

[ece38525-bib-0011] Canham, C. D. , LePage, P. T. , & Coates, K. D. (2004). A neighborhood analysis of canopy tree competition: Effects of shading versus crowding. Canadian Journal of Forest Research, 34, 778–787. 10.1139/x03-232

[ece38525-bib-0012] Cao, J. , Zhang, C. , Zhao, B. , Li, X. , Hou, M. , & Zhao, X. (2018). Seedling density dependence regulated by population density and habitat filtering: Evidence from a mixed primary broad‐leaved Korean pine forest in Northeastern China. Annals of Forest Science, 75, 25. 10.1007/s13595-018-0706-x

[ece38525-bib-0013] Chang, L. W. , Hwong, J. L. , Chiu, S. T. , Wang, H. H. , Yang, K. C. , Chang, H. Y. , & Hsieh, C. F. (2010). Species composition, size‐class structure, and diversity of the Lienhuachih forest dynamics plot in a subtropical evergreen broad‐leaved forest in central Taiwan. Taiwan Journal of Forest Science, 25, 81–95. 10.7075/TJFS.201003.0081

[ece38525-bib-0014] Chen, L. , Comita, L. S. , Wright, S. J. , Swenson, N. G. , Zimmerman, J. K. , Mi, X. , Hao, Z. , Ye, W. , Hubbell, S. P. , Kress, W. J. , Uriarte, M. , Thompson, J. , Nytch, C. J. , Wang, X. , Lian, J. , & Ma, K. (2018). Forest tree neighborhoods are structured more by negative conspecific density dependence than by interactions among closely related species. Ecography, 41, 1114–1123. 10.1111/ecog.03389

[ece38525-bib-0015] Chen, L. , Mi, X. , Comita, L. S. , Zhang, L. , Ren, H. , & Ma, K. (2010). Community‐level consequences of density dependence and habitat association in a subtropical broad‐leaved forest. Ecology Letters, 13, 695–704. 10.1111/j.1461-0248.2010.01468.x 20412278

[ece38525-bib-0016] Chesson, P. (2000). Mechanisms of maintenance of species diversity. Annual Review of Ecology and Systematics, 31, 343–366. 10.1146/annurev.ecolsys.31.1.343

[ece38525-bib-0017] Comita, L. S. , Condit, R. , & Hubbell, S. P. (2007). Developmental changes in habitat associations of tropical trees. Journal of Ecology, 95, 482–492. 10.1111/j.1365-2745.2007.01229.x

[ece38525-bib-0018] Comita, L. S. , & Hubbell, S. P. (2009). Local neighborhood and species’ shade tolerance influence survival in a diverse seedling bank. Ecology, 90, 328–334. 10.1890/08-0451.1 19323215

[ece38525-bib-0019] Comita, L. S. , Muller‐Landau, H. C. , Aguilar, S. , & Hubbell, S. P. (2010). Asymmetric density dependence shapes species abundances in a tropical tree community. Science, 329, 330–332. 10.1126/science.1190772 20576853

[ece38525-bib-0020] Comita, L. S. , Queenborough, S. A. , Murphy, S. J. , Eck, J. L. , Xu, K. , Krishnadas, M. , Beckman, N. , & Zhu, Y. (2014). Testing predictions of the Janzen‐Connell hypothesis: A meta‐analysis of experimental evidence for distance‐ and density‐dependent seed and seedling survival. Journal of Ecology, 102, 845–856. 10.1111/1365-2745.12232 PMC4140603 25253908

[ece38525-bib-0021] Comita, L. , Uriarte, M. , Forero‐Montaña, J. , Kress, W. , Swenson, N. , Thompson, J. , Umaña, M. , & Zimmerman, J. (2018). Changes in phylogenetic community structure of the seedling layer following hurricane disturbance in a human‐impacted tropical forest. Forests, 9, 556. 10.3390/f9090556

[ece38525-bib-0022] Condit, R. (1998). Tropical forest census plots: Methods and results from Barro Colorado Island, Panama and a comparison with other plots. Springer.

[ece38525-bib-0023] Connell, J. H. (1971). On the role of natural enemies in preventing competitive exclusion in some marine animals and in rain forest trees. In P. J. den Boer & G. R. Gradwell (Eds.), Dynamics of populations (pp. 298–313). Centre for Agricultural Publishing and Documentation.

[ece38525-bib-0024] Dormann, C. F. , McPherson, J. M. , Araújo, M. B. , Bivand, R. , Bolliger, J. , Carl, G. , Davies, R. G. , Hirzel, A. , Jetz, W. , Kissling, W. D. , Kühn, I. , Ohlemüller, R. , Peres‐Neto, P. R. , Reineking, B. , Schröder, B. , Schurr, F. M. , & Wilson, R. (2007). Methods to account for spatial autocorrelation in the analysis of species distributional data: A review. Ecography, 30, 609–628. 10.1111/j.2007.0906-7590.05171.x

[ece38525-bib-0025] Du, Y. , Queenborough, S. A. , Chen, L. , Wang, Y. , Mi, X. , Ma, K. , & Comita, L. S. (2017). Intraspecific and phylogenetic density‐dependent seedling recruitment in a subtropical evergreen forest. Oecologia, 184, 193–203. 10.1007/s00442-017-3842-3 28238049

[ece38525-bib-0026] Engelbrecht, B. M. J. , & Kursar, T. A. (2003). Comparative drought‐resistance of seedlings of 28 species of co‐occurring tropical woody plants. Oecologia, 136, 383–393. 10.1007/s00442-003-1290-8 12811534

[ece38525-bib-0027] Harms, K. E. , Condit, R. , Hubbell, S. P. , & Foster, R. B. (2001). Habitat associations of trees and shrubs in a 50‐ha neotropical forest plot. Journal of Ecology, 89, 947–959. 10.1111/j.1365-2745.2001.00615.x

[ece38525-bib-0028] Harms, K. E. , Wright, S. J. , Calderón, O. , Hernández, A. , & Herre, E. A. (2000). Pervasive density‐dependent recruitment enhances seedling diversity in a tropical forest. Nature, 404, 493–495. 10.1038/35006630 10761916

[ece38525-bib-0029] Hood, L. A. , Swaine, M. D. , & Mason, P. A. (2004). The influence of spatial patterns of damping‐off disease and arbuscular mycorrhizal colonization on tree seedling establishment in Ghanaian tropical forest soil. Journal of Ecology, 92, 816–823. 10.1111/j.0022-0477.2004.00917.x

[ece38525-bib-0030] Janzen, D. H. (1970). Herbivores and the number of tree species in tropical forests. The American Naturalist, 104, 501–528. 10.1086/282687

[ece38525-bib-0031] Johnson, D. J. , Beaulieu, W. T. , Bever, J. D. , & Clay, K. (2012). Conspecific negative density dependence and forest diversity. Science, 336, 904–907. 10.1126/science.1220269 22605774

[ece38525-bib-0032] Johnson, D. J. , Bourg, N. A. , Howe, R. , McShea, W. J. , Wolf, A. , & Clay, K. (2014). Conspecific negative density‐dependent mortality and the structure of temperate forests. Ecology, 95, 2493–2503. 10.1890/13-2098.1

[ece38525-bib-0033] Keddy, P. A. (1992). Assembly and response rules: Two goals for predictive community ecology. Journal of Vegetation Science, 3, 157–164. 10.2307/3235676

[ece38525-bib-0034] Kembel, S. W. , Cowan, P. D. , Helmus, M. R. , Cornwell, W. K. , Morlon, H. , Ackerly, D. D. , Blomberg, S. P. , & Webb, C. O. (2010). Picante: R tools for integrating phylogenies and ecology. Bioinformatics, 26, 1463–1464. 10.1093/bioinformatics/btq166 20395285

[ece38525-bib-0035] Kraft, N. J. B. , Cornwell, W. K. , Webb, C. O. , & Ackerly, D. D. (2007). Trait evolution, community assembly, and the phylogenetic structure of ecological communities. The American Naturalist, 170, 271–283. 10.2307/4541080 17874377

[ece38525-bib-0036] Kress, W. J. , Erickson, D. L. , Jones, F. A. , Swenson, N. G. , Perez, R. , Sanjur, O. , & Bermingham, E. (2009). Plant DNA barcodes and a community phylogeny of a tropical forest dynamics plot in Panama. Proceedings of the National Academy of Sciences, 106, 18621–18626. 10.1073/pnas.0909820106 PMC2763884 19841276

[ece38525-bib-0037] Larsen, D. R. , & Speckman, P. L. (2004). Multivariate regression trees for analysis of abundance data. Biometrics, 60, 543–549. 15180683 10.1111/j.0006-341X.2004.00202.x

[ece38525-bib-0038] Lebrija‐Trejos, E. , Wright, S. J. , Hernández, A. , & Reich, P. B. (2014). Does relatedness matter? Phylogenetic density‐dependent survival of seedlings in a tropical forest. Ecology, 95, 940–951. 10.1890/13-0623.1 24933813

[ece38525-bib-0039] Liang, M. , Johnson, D. , Burslem, D. F. R. P. , Yu, S. , Fang, M. , Taylor, J. D. , Taylor, A. F. S. , Helgason, T. , & Liu, X. (2020). Soil fungal networks maintain local dominance of ectomycorrhizal trees. Nature Communications, 11, 2636. 10.1038/s41467-020-16507-y PMC7250933 32457288

[ece38525-bib-0040] Lin, L. , Comita, L. S. , Zheng, Z. , & Cao, M. (2012). Seasonal differentiation in density‐dependent seedling survival in a tropical rain forest. Journal of Ecology, 100, 905–914. 10.1111/j.1365-2745.2012.01964.x

[ece38525-bib-0041] Liu, X. , Liang, M. , Etienne, R. S. , Wang, Y. , Staehelin, C. , & Yu, S. (2012). Experimental evidence for a phylogenetic Janzen‐Connell effect in a subtropical forest. Ecology Letters, 15, 111–118. 10.1111/j.1461-0248.2011.01715.x 22082078

[ece38525-bib-0042] Losos, J. B. (2008). Phylogenetic niche conservatism, phylogenetic signal and the relationship between phylogenetic relatedness and ecological similarity among species. Ecology Letters, 11, 995–1003. 10.1111/j.1461-0248.2008.01229.x 18673385

[ece38525-bib-0043] Lu, J. , Johnson, D. J. , Qiao, X. , Lu, Z. , Wang, Q. , & Jiang, M. (2015). Density dependence and habitat preference shape seedling survival in a subtropical forest in central China. Journal of Plant Ecology, 8, 568–577. 10.1093/jpe/rtv006

[ece38525-bib-0044] Lu, S. Y. , Hwang, L. S. , & Huang, H. H. (2008). Complication of meteorological records for the Lienhuachih for the station 1997–2007. Taiwan Forestry Research Institude.

[ece38525-bib-0045] Mangan, S. A. , Schnitzer, S. A. , Herre, E. A. , Mack, K. M. L. , Valencia, M. C. , Sanchez, E. I. , & Bever, J. D. (2010). Negative plant‐soil feedback predicts tree‐species relative abundance in a tropical forest. Nature, 466, 752–755. 10.1038/nature09273 20581819

[ece38525-bib-0046] Metz, M. R. (2012). Does habitat specialization by seedlings contribute to the high diversity of a lowland rain forest? Journal of Ecology, 100, 969–979. 10.1111/j.1365-2745.2012.01972.x

[ece38525-bib-0047] Metz, M. R. , Sousa, W. P. , & Valencia, R. (2010). Widespread density‐dependent seedling mortality promotes species coexistence in a highly diverse Amazonian rain forest. Ecology, 91, 3675–3685. 10.1890/08-2323.1 21302838

[ece38525-bib-0048] Murphy, S. J. , Wiegand, T. , & Comita, L. S. (2017). Distance‐dependent seedling mortality and long‐term spacing dynamics in a neotropical forest community. Ecology Letters, 20, 1469–1478. 10.1111/ele.12856 28980377

[ece38525-bib-0069] Nakagawa, S. , & Schielzeth, H. (2013). A general and simple method for obtaining R2 from generalized linear mixed‐effects models. Methods in Ecology and Evolution, 4, 133–142. 10.1111/j.2041-210x.2012.00261.x

[ece38525-bib-0049] Newbery, D. M. , & Stoll, P. (2013). Relaxation of species‐specific neighborhood effects in Bornean rain forest under climatic perturbation. Ecology, 94, 2838–2851. 10.1890/13-0366.1 24597229

[ece38525-bib-0050] Oksanen, J. , Blanchet, F. G. , Friendly, M. , Kindt, R. , Legendre, P. , McGlinn, D. , Minchin, P. R. , O'Hara, R. B. , Simpson, G. L. , Solymos, P. , Stevens, M. H. H. , Szoecs, E. , & Wagner, H. (2020). vegan: Community Ecology Package. R package version 2.5‐7. https://CRAN.R‐project.org/package=vegan

[ece38525-bib-0051] Paine, C. E. T. , Norden, N. , Chave, J. , Forget, P.‐M. , Fortunel, C. , Dexter, K. G. , & Baraloto, C. (2011). Phylogenetic density dependence and environmental filtering predict seedling mortality in a tropical forest. Ecology Letters, 15, 34–41. 10.1111/j.1461-0248.2011.01705.x 22004454

[ece38525-bib-0052] Paradis, E. , Claude, J. , & Strimmer, K. (2004). APE: Analyses of phylogenetics and evolution in R language. Bioinformatics, 20, 289–290. 10.1093/bioinformatics/btg412 14734327

[ece38525-bib-0053] Peters, H. A. (2003). Neighbour‐regulated mortality: The influence of positive and negative density dependence on tree populations in species‐rich tropical forests. Ecology Letters, 6, 757–765. 10.1046/j.1461-0248.2003.00492.x

[ece38525-bib-0054] Piao, T. , Comita, L. , Jin, G. , & Kim, J. (2013). Density dependence across multiple life stages in a temperate old‐growth forest of northeast China. Oecologia, 172, 207–217. 10.1007/s00442-012-2481-y 23053238 PMC3627022

[ece38525-bib-0055] Pu, X. , & Jin, G. (2018). Conspecific and phylogenetic density‐dependent survival differs across life stages in two temperate old‐growth forests in Northeast China. Forest Ecology and Management, 424, 95–104. 10.1016/j.foreco.2018.04.055

[ece38525-bib-0056] Pu, X. , Zhu, Y. , & Jin, G. (2017). Effects of local biotic neighbors and habitat heterogeneity on seedling survival in a spruce‐fir valley forest, northeastern China. Ecology and Evolution, 7, 4582–4591. 10.1002/ece3.3030 28690788 PMC5496565

[ece38525-bib-0070] R Core Team . (2021). R: A language and environment for statistical computing. R Foundation for Statistical Computing. https://www.R‐project.org/

[ece38525-bib-0057] Record, S. , Kobe, R. K. , Vriesendorp, C. F. , & Finley, A. O. (2016). Seedling survival responses to conspecific density, soil nutrients, and irradiance vary with age in a tropical forest. Ecology, 97, 2406–2415. 10.1002/ecy.1458 27859074

[ece38525-bib-0058] Sanderson, M. J. (2003). r8s: Inferring absolute rates of molecular evolution and divergence times in the absence of a molecular clock. Bioinformatics, 19, 301–302. 10.1093/bioinformatics/19.2.301 12538260

[ece38525-bib-0059] Simard, S. W. , Perry, D. A. , Jones, M. D. , Myrold, D. D. , Durall, D. M. , & Molina, R. (1997). Net transfer of carbon between ectomycorrhizal tree species in the field. Nature, 388, 579–582. 10.1038/41557

[ece38525-bib-0060] Song, X. , Johnson, D. J. , Cao, M. , Umaña, M. N. , Deng, X. , Yang, X. , Zhang, W. , & Yang, J. (2018). The strength of density‐dependent mortality is contingent on climate and seedling size. Journal of Vegetation Science, 29, 662–670. 10.1111/jvs.12645

[ece38525-bib-0061] Stamatakis, A. , Hoover, P. , & Rougemont, J. (2008). A rapid bootstrap algorithm for the RAxML web servers. Systematic Biology, 57, 758–771. 10.1080/10635150802429642 18853362

[ece38525-bib-0062] Webb, C. O. (2000). Exploring the phylogenetic structure of ecological communities: An example for rain forest trees. The American Naturalist, 156, 145–155. 10.1086/303378 10856198

[ece38525-bib-0063] Webb, C. O. , Gilbert, G. S. , & Donoghue, M. J. (2006). Phylodiversity‐dependent seedling mortality, size structure, and disease in a Bornean rain forest. Ecology, 87, 123–131. 10.1890/0012-9658(2006)87[123:PSMSSA]2.0.CO;2 16922308

[ece38525-bib-0064] Wills, C. , & Green, D. R. (1995). A genetic herd‐immunity model for the maintenance of MHC polymorphism. Immunological Reviews, 143, 263–292. 10.1111/j.1600-065X.1995.tb00679.x 7558080

[ece38525-bib-0065] Wright, J. (2002). Plant diversity in tropical forests: A review of mechanisms of species coexistence. Oecologia, 130, 1–14. 10.1007/s004420100809 28547014

[ece38525-bib-0066] Wu, B.‐W. , Gao, C. , Chen, L. , Buscot, F. , Goldmann, K. , Purahong, W. , Ji, N.‐N. , Wang, Y.‐L. , Lü, P.‐P. , Li, X.‐C. , & Guo, L.‐D. (2018). Host phylogeny is a major determinant of fagaceae‐associated ectomycorrhizal fungal community assembly at a regional scale. Frontiers in Microbiology, 9, 1–12. 10.3389/fmicb.2018.02409 30364168 PMC6191505

[ece38525-bib-0067] Wu, J. , Swenson, N. G. , Brown, C. , Zhang, C. , Yang, J. , Ci, X. , Li, J. , Sha, L. , Cao, M. , & Lin, L. (2016). How does habitat filtering affect the detection of conspecific and phylogenetic density dependence? Ecology, 97, 1182–1193. 10.1890/14-2465.1 27349095

[ece38525-bib-0068] Zhu, Y. , Comita, L. S. , Hubbell, S. P. , & Ma, K. (2015). Conspecific and phylogenetic density‐dependent survival differs across life stages in a tropical forest. Journal of Ecology, 103, 957–966. 10.1111/1365-2745.12414

