# Supplementary material for: Seedling survival simultaneously determined by conspecific, heterospecific, and phylogenetically related neighbors and habitat heterogeneity in a subtropical forest in Taiwan
Source: Ecol Evol. 2022 Jan 12;12(1):e8525. doi: 10.1002/ece3.8525 (PMC8809428; doi:10.1002/ece3.8525)

FIGURE S1. The coefficient estimates and 95% confidence intervals of community-level mean relationships between focal seedlings at different neighborhood scales (5 m, 10 m, 20 m) on the Lienhuachih Forest Dynamic Plot, Taiwan. Coefficient estimates above and below unity indicate positive and negative effects, respectively. The black circles indicate significant effects ($P < 0.05$), and white circles mean no significance.

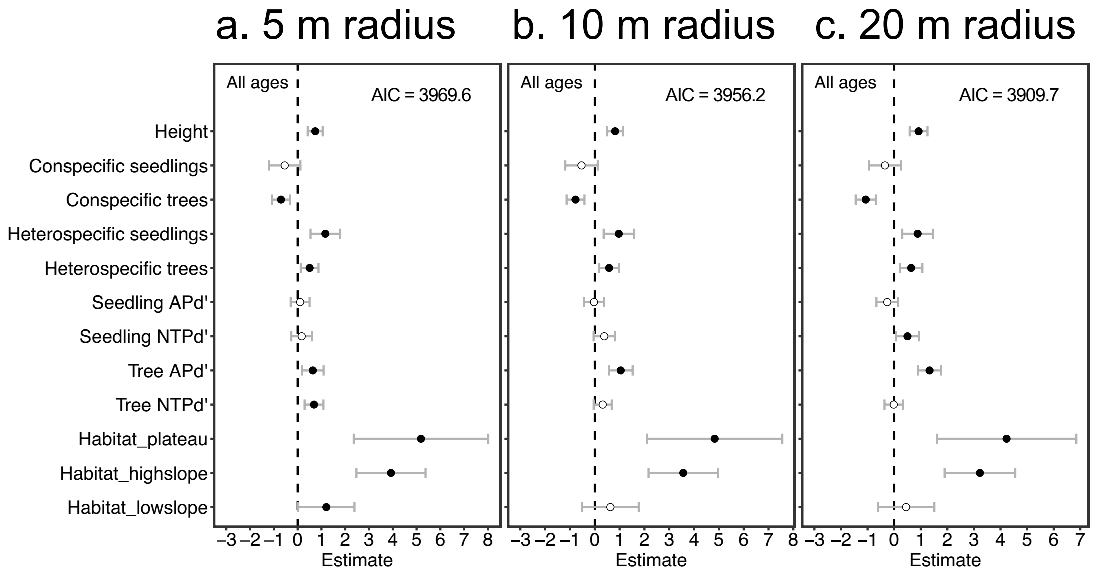

FIGURE S2. Multivariate regression tree (MRT) for the tree species composition against micro-topographic factors in the Lienhuachih Forest Dynamics Plot, Taiwan. The information above each node denotes the selected factor and rule for the division of the quadrats. Numbers in parentheses below each node and terminal give the number of quadrats in that branch. The length of the branch is proportional to the variation explained by tree splitting.

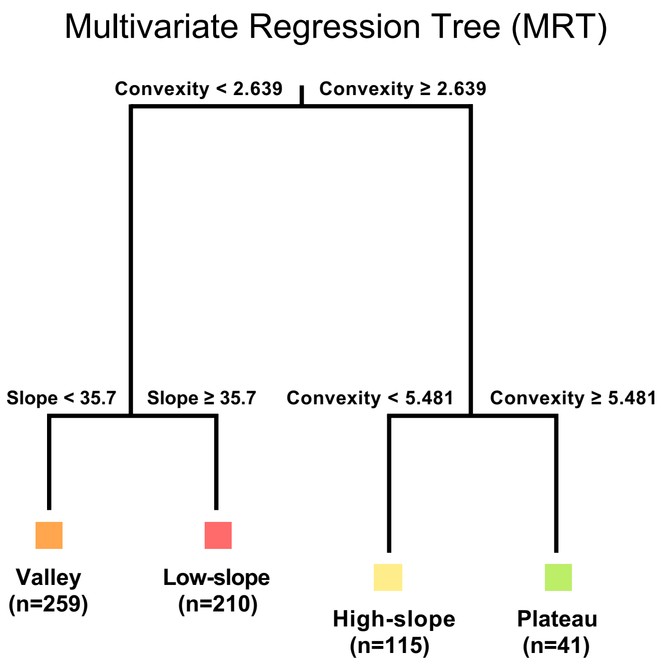

FIGURE S3. Map of the habitat classified by the multivariate regression tree (MRT) within the 25-ha Lienhuachih Forest Dynamics Plot. The four different habitats were shown by different colors and the plot was divided to 625 quadrats (20 m × 20 m).

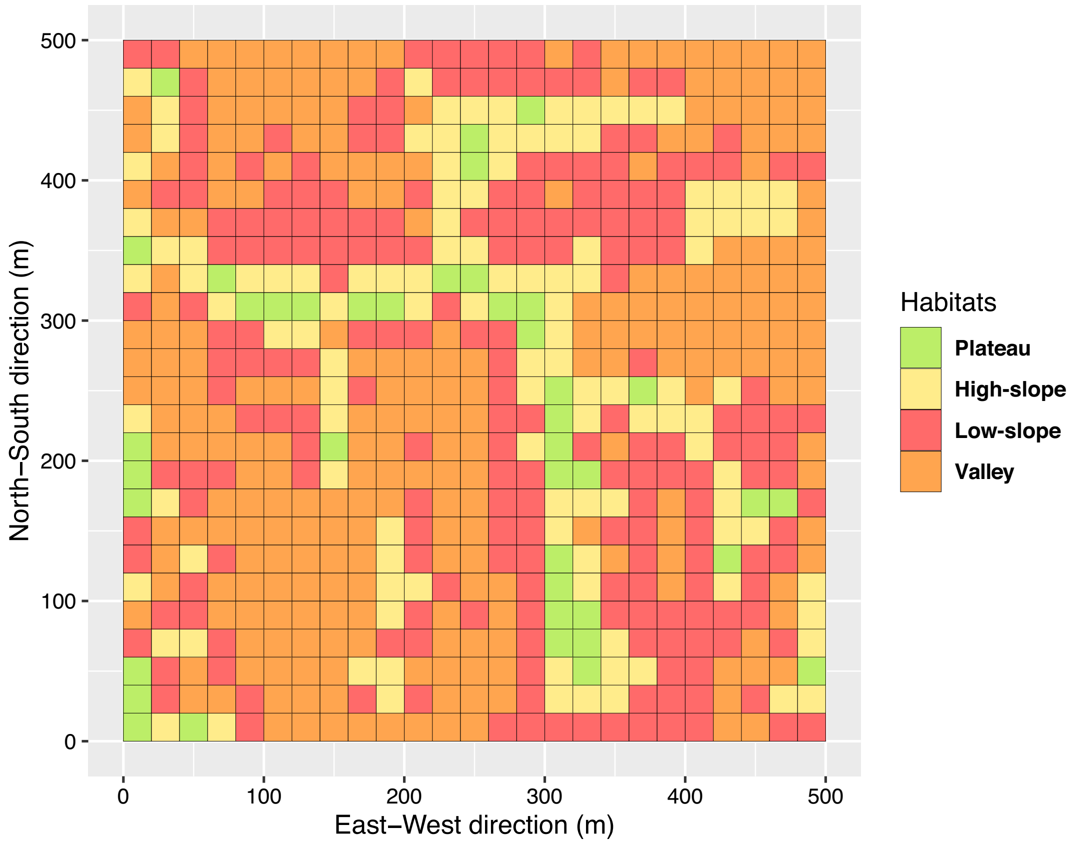

FIGURE S4. The differences in mean levels of soil moisture among four habitat types in the Lienhuachih Forest Dynamics Plot, Taiwan. The differences among habitats were estimated by Tukey's HSD test. The black circles indicate significant effects ($P < 0.05$), and white circles mean no significance.

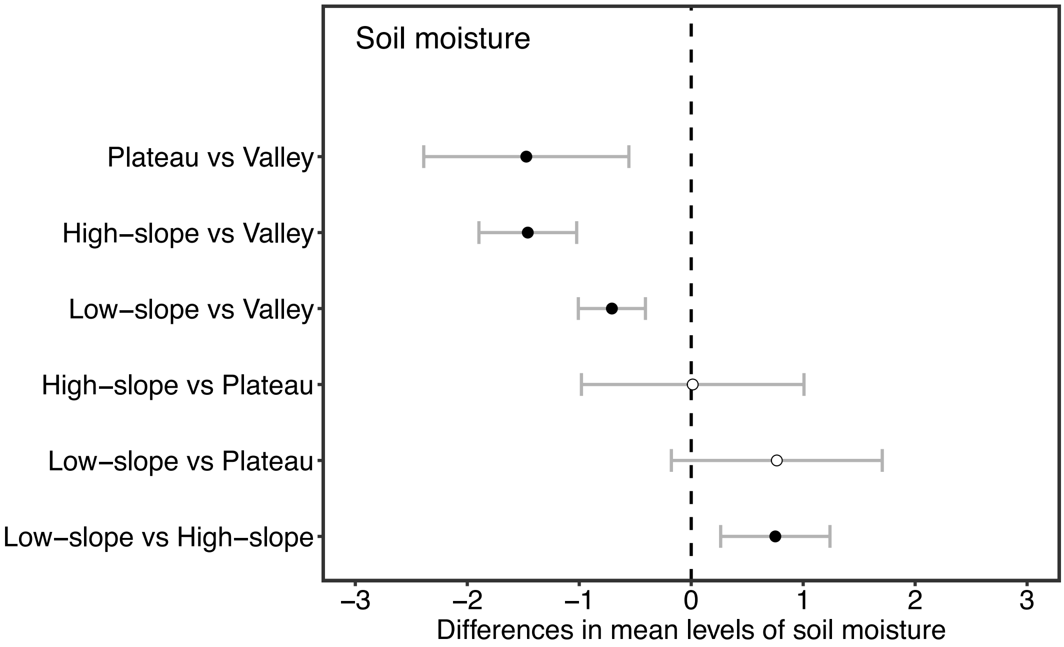

Supplement: Supplementary file 1 — Supplementary Material [file ECE3-12-e8525-s001.pdf]
